# A Child One and a Half Years Old with a Shingle (4 Penny) Nail in Its Windpipe—Some Other Cases of Foreign Bodies in the Trachea and Esophagus Reported

**Published:** 1901-12

**Authors:** Wm. Lewis Bullard

**Affiliations:** Columbus, Ga., Member of the American Medical Association; The Georgia Medical Association ; The Tri-State Medical Association of Georgia, Alabama and Tennessee; President of the Columbus Board of Health; Vice-President of the American Congress of Tuberculosis


### A CHILD ONE AND A HALF YEARS OLD WITH A SHINGLE (4 PENNY) NAIL IN ITS WINDPIPE—SOME OTHER CASES OF FOREIGN BODIES IN THE TRACHEA AND ESOPHAGUS REPORTED.

BY WM. LEWIS BULLARD, M.D., COLUMBUS, GA.,

Member of the American Medical Association; The Georgia Medical Association; The Tri-State Medical Association of Georgia, Alabama and Tennessee; President of the Columbus Board of Health; Vice-President of the American Congress of Tuberculosis.

In the February number of the *Alabama Medical and Surgical Age,* 1893, may be found "Cockle-burr in the Air Passage of a Child Successfully Removed by an Intra-Laryngeal Operation by W. L. Bullard, M.D., Columbus, Georgia." Part of that report is: "That I would be able to deviate from the general rule, that is to say, remove the corpus alienum by an intra-laryngeal operation instead of tracheotomy, as advised by most text-books on surgery and practiced by the most eminent surgeons. I hastened to cocainize the pharynx and larynx with a solution of cocain, and with the co-operation of the little patient, it was not long before I had the burr safely in the grasp of a Steerk's tube forceps and readily extracted it, to the admiration of Dr. Sapp and indescribable relief to the patient and the father of the child. With some advice as to treatment should the laryngeal inflammation not soon heal or subside, the little hero was put on the first train and carried to his home, instead of having to remain in the city for a week, perhaps, had I performed either laryngotomy or tracheotomy." Hamilton (1) says: "In short, we have very few resources in this unfortunate class of accidents except a surgical operation. If the object for which we are to search is lodged in the ventricles of the larynx, the operation of laryngotomy is to be performed."

Agnew (2) advises, in that most classical work of his on surgery, that "When it can be determined that the body is located in one of the ventricles of the larynx laryngotomy is to be preferred." The Atlanta Society of Medicine reports (3) the following: "Dr. ——— reported two cases of tracheotomy for foreign bodies. One, a girl of 8 years old, swallowed a knitting-needle bent in the shape of a hairpin. Cut down and found it without much difficulty. The second swallowed a cockle-burr four days previously; performed tracheotomy. Could not find the foreign body; cut two more rings of the trachea, kept wound open and the cockle-burr came out four days after the operation. I mention the above only as evidence to show that the tendency is to remove foreign bodies from the air passages by an external operation as tracheotomy and laryngotomy, ignoring intra-laryngeal efforts, which according to my mind and experience (if the patient is in no immediate danger), should always be more diligently practiced as the most preferable method, and if found impracticable, then resort to one of the external operations." It is nearly ten years since the above was written, and yet I am willing to repeat it as good advice, for I occasionally see reported cases of foreign bodies in the trachea, operated upon without even making a laryngoscophical examination, notwithstanding the advancement in laryngological surgery, so ably advanced by numerous laryngologists.

Since reporting the above case I have frequently been consulted regarding foreign bodies in the throat. The last one about one week ago. A child one and a half years old, of Butler, Ga., referred to me by Dr. Edwards and was brought by its mother and father, both of whom were very much alarmed, asserting that the child had a nail in its throat. When I inquired if they knew positively that it was a nail they answered "no," but said that the child's sister, who at the time was nursing the child, said it was a nail. Two physicians had seen the case (one an eye, ear and nose specialist) both diagnosing a foreign body in the trachea, and advised that the case be brought to me. I learned that the accident happened five days previously. At times it would have paroxisms of the most violent coughing, which would last for several minutes, then a stage of quiesence, lasting for an hour or more. The cough was croupy, indicative of laryngitis and bronchitis. After a thorough cocainizing, together with adrenaline spray to the pharynx and larynx a laryngoscopic examination revealed an unobstructed larynx and glottis, which convinced us that tracheotomy was the only resource for relief. This we tendered to the child's parents, who after another day of suspense asked to have the operation performed. The patient was soon chloroformed by Dr. Gill, of this city, and with the help of Mr. Amory Dexter, a three-year student at the "Jeff," I did the low operation, and without difficulty, removed a shingle (4 penny) nail. The trachea, muscles and integument were sutured separately, and the wound readily healed, and in six days patient returned home fully recovered.

F. B., child, two years old, brought to me by its mother, accompanied by Dr. Rodges, the family physician, of Reynolds, Ga., with a watermelon seed in the windpipe. A laryngoscopic examination assured us that the foreign substance was below the glottis; besides we could hear the up and down movement of the seed in the trachea during inspiration and expiration. Tracheotomy was advised and readily consented to. Patient was soon chloroformed by Dr. Rodges, and with the competent assistance of Dr. Geo. J. Grimes, of this city, I, without difficulty, opened the trachea and almost instantly on expiration the seed was expelled, striking the ceiling of my office. Wound was dressed in the usual way and soon healed, patient returning home six days after the operation.

S. C., age eight years, a bright little girl, possessed of no small amount of reason, was brought to me some months ago for having swallowed a pin. The accident had just happened a few hours before. I cocainized the pharynx and larynx and with the laryngoscope readily located the pin, firmly transfixed horizontally in the ventricles of the larynx. Forceps were introduced intralaryngeally, the pin grasped in its middle, and with some difficulty, was extracted, having been bent almost double on account of the force with which it required to dislodge it. The ends of the pin cut either side of the larynx during the extraction, but this soon subsided and in a few days the voice regained its normal resonance and patient dismissed as well.

Two years ago I was consulted by a lady who claimed to have something in her throat. Said that her husband was sick with slow fever, and that she had made him some chicken soup, and in tasting it to find out when suitable for the fever-stricken patient something lodged in her throat. The family physician examined the throat microscopically, could see nothing, and advised that in a day or so the irritation would have passed away. This prognosis did not materialize, so in three or four days I saw her. From her description I thought the trouble to be in the esophagus. The use of the laryngoscope revealed nothing foreign in the pharynx or larynx. I then introduced into the stomach a bristle probang with the hope that I might dislodge the disturbing element, either going or coming, so to speak, and sure enough, on withdrawing the instrumant, attached thereto was a fractured bone, one and a half inches long. Patient was greatly delighted, and also somewhat embarrassed. I suggested that posibly it was best that she *only* "tasted" the soup. The irritation from the piece of splintered bone, together with its extraction, caused no little esophagitis with painful deglutition and dysphagia of several days duration. The whole neck was considerably swollen. After a few days, however, with simple treatment she fully recovered.

Another case, Mr. W. B., aged 60, came to me at night, saying that he had something "sticking in his throat," adding "that he had gone into a restaurant, took a drink, after which a mouthful of chicken salid, and necessarially the spicula of bone or whatever it might be, must be very small." An examination of the pharynx and larynx revealed nothing foreign, so I introduced a probang into the esophagus, thinking possibly I might dislodge the obstructing body, which could be felt during the passage of the instrument about midway between the pharynx and stomach. On the withdrawal of the instrument the foreign body was brought nearer towards the pharynx. The instrument was again introduced, and on its withdrawal, a large piece of the breastbone of a chicken was removed. The patient was very much excited and appeared to be in great distress. Its removal gave complete relief and the next morning he was at his usual place of business, showing no symptom of his trouble the night before.

I have several other cases, but they are similar to the above and to report them would be more of a reiteration; then, also, the motive which prompts this report is to show that while frequently the majority operations are required for the removal of foreign bodies from the windpipe and gullet, yet not infrequently the same may be accomplished by intra-larynaeal and intra-esophageal methods which, however, in unreasonable children with foreign bodies in the glottis, I'll admit, are more delicate and requires more *knack* and *tact* to successfully perform than either tracheotomy or esophagotomy, nevertheless, it might be more satisfactory and safer for the patient.

